# High HIV Incidence and Sexual Behavior Change among Pregnant Women in Lilongwe, Malawi: Implications for the Risk of HIV Acquisition

**DOI:** 10.1371/journal.pone.0039109

**Published:** 2012-06-29

**Authors:** Maria A. Keating, Gloria Hamela, William C. Miller, Agnes Moses, Irving F. Hoffman, Mina C. Hosseinipour

**Affiliations:** 1 University of North Carolina Project, Lilongwe, Malawi; 2 Department of Obstetrics and Gynecology, Drexel University College of Medicine, Philadelphia, Pennsylvania, United States of America; 3 Department of Medicine, University of North Carolina School of Medicine, Chapel Hill, North Carolina, United States of America; University of Cape Town, South Africa

## Abstract

**Background:**

HIV incidence is higher among pregnant women than their non-pregnant counterparts in some sub-Saharan African settings. Our aims were (1) to estimate HIV incidence during pregnancy and (2) to compare sexual activity between pregnant, postpartum, and non-pregnant women.

**Methods:**

We examined a retrospective cohort of 1087 women to identify seroconverters using antenatal and labor ward HIV test results. We also conducted a cross-sectional survey, including a quantitative questionnaire (n = 200) and in-depth interviews (n = 20) among women in early pregnancy, late pregnancy, postpartum, and non-pregnancy. Outcomes included measures of sexual activity, reported spouse’s risky behavior, and beliefs about abstinence.

**Results:**

11 of 1087 women seroconverted during pregnancy yielding a 1% seroconversion risk and an incidence rate of 4.0/100 person years (95% CI 2.2–7.2). The reported sexual activity of the early pregnancy and non-pregnancy groups was similar, but significantly higher than the late pregnancy and postpartum groups (p<0.001). During pregnancy, sex acts decreased as gestation increased (p = 0.001). There was no reported difference in the spouse’s risky behavior. Most women believed that sex should cease between the 6^th^ and 8^th^ month of pregnancy and should not resume until 6 months postpartum. Some talked about conflict between their cultural obligation to abstain and fear of HIV infection if their spouses find other partners.

**Conclusions:**

HIV incidence is high among pregnant women in Malawi, and sexual activity decreases during pregnancy and postpartum. Pregnant women need to be informed of their increased risk for HIV and the importance of using condoms throughout pregnancy and the postpartum period.

## Introduction

In some sub-Saharan African settings, HIV incidence is higher among pregnant women than their non-pregnant counterparts [1]–[4]. The greater risk of HIV acquisition may be explained by both biological and behavioral factors [2], [5].

Sexual activity of the pregnant woman decreases during pregnancy and the postpartum period, but that decrease may lead to riskier behavior among male spouses [5]–[9]. In West Africa, male partners of pregnant and postpartum women have increased extramarital sexual contacts. Biologically, increased risk may be due to increases in the primary endocervical receptor for HIV-1, CCR5, associated with the progesterone-predominant state of pregnancy [10].

In Malawi, primary prevention of HIV infection among women of child-bearing age is the first prong of national prevention of mother-to-child transmission (PMTCT) guidelines [11]. To achieve this goal, the factors that put pregnant and postpartum women at risk of HIV acquisition must be identified to ensure appropriate interventions are integrated into PMTCT programs. In this study, we sought to estimate HIV incidence during pregnancy and to describe sexual behavior among pregnant, postpartum, and non-pregnant women in Lilongwe, Malawi. We hypothesized that (1) HIV incidence is high among pregnant women; (2) sexual activity between women and their spouses decreases during pregnancy, particularly late pregnancy, and the postpartum period; and (3) women perceive increased risky behavior in their spouses during late pregnancy and the postpartum period.

## Methods

### Study Setting

All study activities were conducted at Bwaila Hospital, the largest government antenatal facility in Lilongwe District, Malawi. Bwaila provides antenatal, labor & delivery, postnatal, and family planning services, including antenatal PMTCT and labor ward HIV testing. Using an “opt-out” method for HIV testing, 99% of women are tested at their first antenatal visit. HIV tests are also routinely performed at the labor ward for (1) women with unknown HIV status and (2) women with a known negative test ≥3 months prior. Serial HIV testing is performed first with the Determine HIV-1/2 assay (99.8% sensitivity, 99.4% specificity) and then, if positive, with the Uni-Gold Recombigen HIV assay (98.5% sensitivity, 99.5% specificity) following Malawi’s national protocol [12]. All antenatal, labor ward, postnatal, and family planning visits are recorded in standard Malawi Ministry of Health registers and each woman is assigned a unique registration number, which can be used to trace them at subsequent visits. The registers are subsequently entered into Access databases with each service having its own database.

### HIV Incidence

In order to estimate HIV seroconversion risk and incidence rate during pregnancy, we reviewed the antenatal and labor & delivery databases to identify women who attended Bwaila antenatal clinic and delivered at the labor ward. We compared antenatal and labor ward HIV results of those women to identify seroconverters. We matched records between the antenatal and labor & delivery databases both manually and systematically using Stata. Because not all women had registration numbers recorded, we matched using both registration numbers and demographic characteristics unlikely to change during pregnancy to ensure accurate matches while accounting for potential data inconsistencies. All women whose records could be matched between the antenatal clinic and labor ward were included in this calculation without any exclusion criteria.

Manual matches included the following: (1) first/last name matches with matching registration numbers (2) first/last name matches who matched on ≥4 of the following criteria: age ±1 year, gravidity, parity, registration number ±1 digit, home location, or positive HIV result. Systematic matches were identified with Stata starting with the most stringent criteria and proceeding to less stringent criteria as follows: (1) first/last name matches with matching registration numbers (2) last name matches with matching registration numbers (3) closely matching names with matching registration numbers (4) first/last name matches with matching age and location (5) first/last name matches with matching age, gravidity, and parity. Given the unreliability of pregnancy dating and the potential for a change in those dates, we did not use gestational age for matching. On the other hand, a positive HIV result at the first antenatal visit would still be positive upon admission to the labor ward so this was a reliable way of matching the same person over time.

The list of manual and systematic matches was merged and duplicates were removed. Using the final list of matches, each woman’s antenatal and labor ward HIV results were compared to identify those who seroconverted between the first antenatal visit and labor ward admission.

### Sexual Behavior Assessment

We also conducted a cross-sectional, mixed methods survey using both a quantitative questionnaire and in-depth interviews to assess sexual behavior among four groups of women: early pregnancy, late pregnancy, postpartum, and non-pregnancy. We recruited women attending the antenatal, postnatal, and family planning clinics at Bwaila Hospital.

Women waiting for their clinic visits were screened for study eligibility using systematic sampling in which every fourth woman was approached. Women were either pregnant, postpartum or had given birth within the last 5 years. Eligibility criteria included age between 18 and 30 and being married so as to represent the general characteristics of the Bwaila clinic population. Additionally, since our questionnaire required knowledge of a male partner’s behavior, we required women to be living with their spouse during the last 3 months. The four groups were defined as follows: early pregnancy included women at antenatal visits who were at 25 weeks gestation or less, late pregnancy included women at greater than 25 weeks gestation, postpartum included women at postpartum visits who were 6 weeks or less since delivery, and non-pregnancy included women at family planning visits who were between 3 months and 5 years since their last delivery.

We obtained written consent from each participant. Two interviewers, who were trained, bilingual, female Malawians, conducted private, face-to-face interviews in Chichewa. Each participant’s oral responses to the questionnaire were recorded by the interviewer. All consents, questionnaires, and question guides were translated into Chichewa and then back translated into English to ensure clarity of meaning. Demographic information and HIV status were obtained from each individual’s health passport or the clinic register to verify that information.

This study was approved by the National Health Sciences Research Committee of Malawi and the Office of Human Research Ethics of the University of North Carolina at Chapel Hill.

#### (1) Quantitative interviews

Participants provided information about demographics, HIV status, sexual activity with their husband and any other sexual partners, sexually transmitted infection (STI) symptoms, and their husband’s behavior. We conducted a brief pilot study of the questionnaire in the first week of data collection and then revised it for clarity.

The quantitative data were entered into a Microsoft Access database using double data entry. Discrepancies were corrected. Stata/IC 11.0 (StataCorp, College Station, Texas) was used for statistical analyses. Categorical data were compared using Fisher’s exact tests to detect any difference between the study groups. Continuous data were compared using Kruskal Wallis, Wilcoxan rank-sum, Pearson’s correlation coefficient, and t-tests as appropriate. A Ρ value less than 0.05 was considered statistically significant.

#### (2) In-depth interviews

Interviews were audio-taped with participants’ permission. Participants provided information about demographics, HIV status, sexual behavior, their husband’s behavior, and beliefs about sex and abstinence during pregnancy.

The in-depth interviews were simultaneously translated and transcribed into English by the interviewers. Quality control was ensured through review of each transcript and clarification of any translation or content issues. Deductive codes, derived from the research questions and interview guides, were manually applied to the transcripts. Inductive codes were then created based on key concepts from the data. Summaries were created for each code that identified sub-themes and recurrent patterns. Qualitative analyses were done collaboratively by a US medical doctor and a Malawian social scientist.

## Results

### HIV Incidence

**Figure 1:**
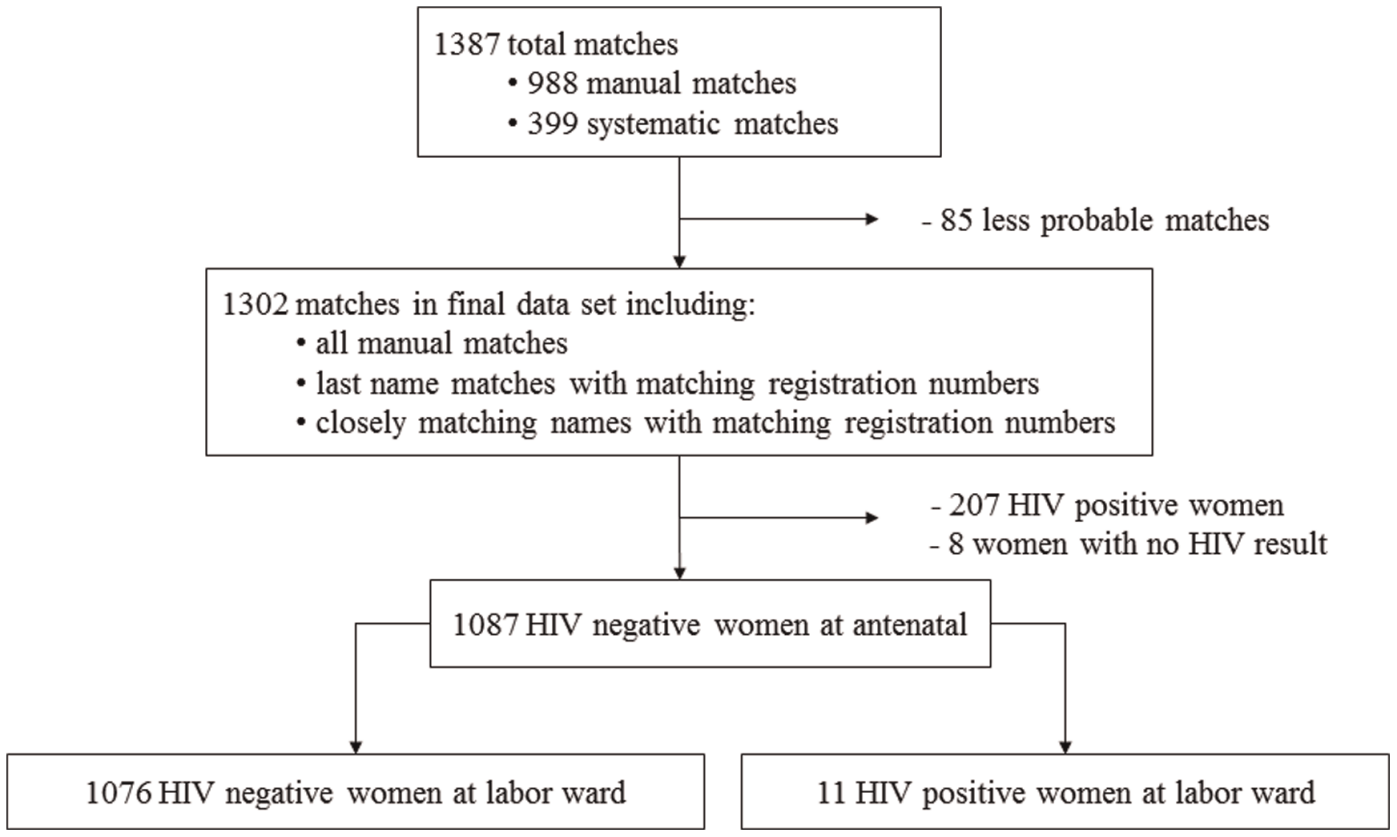
Matching schematic for HIV incidence calculation in the antenatal population at Bwaila Hospital. Antenatal and labor ward records were matched manually and systematically resulting in 1302 final matches whose antenatal and labor ward HIV test results were compared. Of 1087 HIV negative women at the initial antenatal test, 11 tested HIV positive at the labor ward indicating a seroconversion risk of 1% during pregnancy.

Among 1387 women who both (1) attended Bwaila antenatal clinic and (2) delivered at Bwaila labor ward between January and October 2009, we found 1302 (94%) definite matches but only 1294 (93%) had a recorded antenatal HIV result (Figure 1). Of these, 207 (16%) women tested HIV positive at their first antenatal visit, which occurred, on average, at 25 weeks gestation. Among the 1087 women with negative initial HIV tests, 11 seroconverted during pregnancy yielding a 1.0% (11/1087) seroconversion risk and an incidence rate of 4.0 per 100 person years (95% confidence interval (CI) 2.2–7.2). Women with incident HIV infection tended to be older (median age 26 years versus 23 years in non-seroconverters. p = 0.0517), have greater gravidity (median 3 versus 2 in non-seroconverters, p = 0.1034), and have infants with smaller birthweights (median of 2.6 kg versus 3.0 kg in non-seroconverters, p = 0.0806).

### Sexual Behavior

Between January and July 2010, we screened 248 women for study eligibility at the Bwaila antenatal, postnatal, and family planning clinics; 17 women were ineligible (5 were >30 years, 3 were <18 years, 5 were single, and 4 were not living with their spouse) and 11 refused participation. We enrolled 220 participants, 50 in each of the four quantitative groups, and 20 for the in-depth interviews. The groups had similar demographics (Table 1). Overall, the mean age of participants in the quantitative groups was 22.9 (SD 3.6) and the prevalence of HIV was 12.5%. Most of the participants were Christian (85.5%), had primary education or less (63.5%), and were housewives (72.5%).

**Table 1:** Characteristics of quantitative groups and in-depth interview group.

		Early Pregnancyn (%)	Late Pregnancyn (%)	Postpartumn (%)	Non-Pregnancyn (%)	Quantitative Totaln (%)	In-depth Interviewsn (%)
**Age in years**	**18–21**	20 (40%)	23 (46%)	22 (44%)	18 (36%)	83 (41.5%)	7 (35%)
	**22–25**	16 (32%)	22 (44%)	15 (30%)	16 (32%)	69 (34.5%)	11 (55%)
	**26–30**	14 (28%)	5 (10%)	13 (26%)	16 (32%)	48 (24%)	2 (10%)
**HIV Status**	**Positive**	7 (14%)	8 (16%)	4 (8%)	6 (12%)	25 (12.5%)	3 (15%)
	**Negative**	42 (84%)	42 (84%)	45 (90%)	44 (88%)	173 (86.5%)	17 (85%)
	**Not tested**	1 (2%)	0 (0%)	1 (2%)	0 (0%)	2 (1%)	0 (0%)
**Spouse’s HIV Status**	**Positive**	4 (8%)	4 (8%)	2 (4%)	4 (8%)	14 (7%)	1 (5%)
	**Negative**	26 (52%)	31 (62%)	38 (76%)	32 (64%)	127 (63.5%)	14 (70%)
	**Unknown**	2 (4%)	2 (4%)	2 (4%)	3 (6%)	9 (4.5%)	0 (0%)
	**Not tested**	18 (36%)	13 (26%)	8 (16%)	11 (22%)	50 (25%)	5 (25%)
**Gravidity**	**1**	16 (32%)	20 (40%)	18 (36%)	14 (28%)	68 (34%)	5 (25%)
	**2**	14 (28%)	18 (36%)	13 (26%)	18 (36%)	63 (31.5%)	6 (30%)
	**3**	13 (26%)	6 (12%)	13 (26%)	11 (22%)	43 (21.5%)	7 (35%)
	**4–7**	7 (14%)	6 (12%)	6 (12%)	7 (14%)	26 (13%)	2 (10%)
**Education**	**None**	3 (6%)	1 (2%)	3 (6%)	0 (0%)	7 (3.5%)	1 (5%)
	**Primary**	25 (50%)	34 (68%)	29 (58%)	32 (64%)	120 (60%)	13 (65%)
	**Secondary**	20 (40%)	14 (28%)	17 (34%)	17 (34%)	68 (34%)	5 (25%)
	**Post- Secondary**	2 (4%)	1 (2%)	1 (2%)	1 (2%)	5 (2.5%)	1 (5%)
**Religion**	**Christian**	43 (86%)	41 (82%)	44 (88%)	43 (86%)	171 (85.5%)	19 (95%)
	**Muslim**	7 (14%)	9 (18%)	6 (12%)	6 (12%)	28 (14%)	1 (5%)
	**None**	0 (0%)	0 (0%)	0 (0%)	1 (2%)	1 (0.5%)	0 (0%)
**Housing**	**Metal Roof**	45 (90%)	41 (82%)	43 (86%)	45 (90%)	174 (87%)	19 (95%)
	**Thatched Roof**	5 (10%)	9 (18%)	7 (14%)	5 (10%)	26 (13%)	1 (5%)
**Employment**	**Housewife**	36 (72%)	37 (74%)	36 (72%)	36 (72%)	145 (72.5%)	16 (80%)
	**Other**	14 (28%)	13 (26%)	14 (28%)	14 (28%)	55 (27.5%)	4 (20%)
**Spouse’s Employment**	**Business**	22 (44%)	19 (38%)	18 (36%)	19 (38%)	78 (39%)	10 (50%)
	**Other**	28 (56%)	31 (62%)	32 (64%)	31 (62%)	122 (61%)	10 (50%)

**Figure 2:**
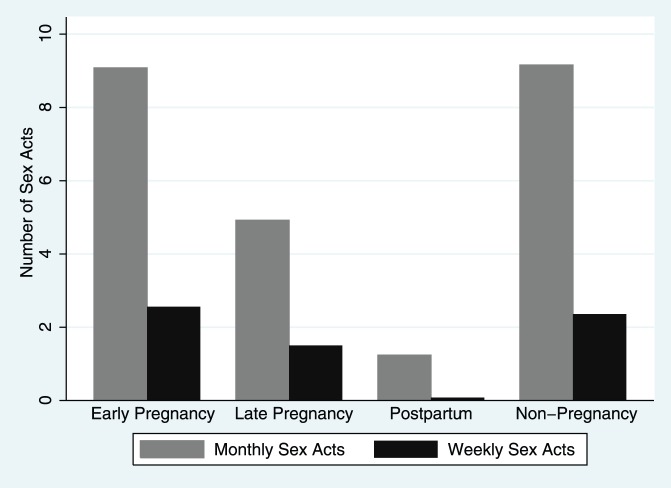
Average Monthly and Weekly Sex Acts among early pregnancy, late pregnancy, postpartum, and non-pregnant study groups. Early pregnancy and non-pregnancy sexual activity was similar and significantly exceeded that of the late pregnancy and postpartum groups when comparing both weekly and monthly sex acts.

**Figure 3:**
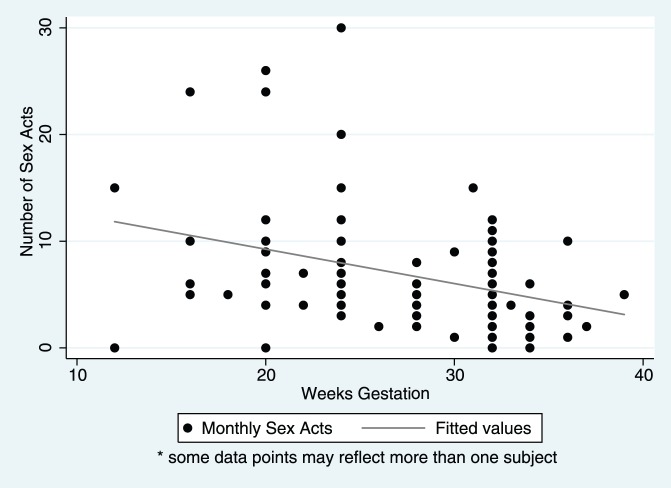
Monthly Sex Acts by Weeks Gestation. Comparing the monthly sex acts of all pregnant participants at various weeks gestation, sexual activity significantly declined as pregnancy advanced with Pearson’s correlation coefficient of −0.322 and p value of 0.001.

Reported sexual activity, by all measures, was similar in early pregnancy and non-pregnancy, but decreased in late pregnancy and almost completely ceased during the postpartum period (Table 2). The reported number of sex acts in the last month in the early pregnancy and non-pregnancy groups was similar with averages of 9.1 and 9.2 sex acts respectively, but significantly exceeded the late pregnancy (4.9 sex acts) and postpartum (1.2 sex acts) monthly averages. Similarly, the weekly averages for the early pregnancy (2.6 sex acts) and non-pregnancy (2.4 sex acts) groups exceeded those of the late pregnancy (1.5 sex acts) and postpartum (0.1 sex acts) groups (Figure 2). In the postpartum group, 92% (46/50) reported no sex in the last week, and 62% (31/50) reported no sex in the last month. In pair-wise comparisons of each measure of sexual activity, all of the groups significantly differed except for the early pregnancy and non-pregnancy groups (p values ranged from 0.1369 to 0.8162). Considering the pregnant women alone, the number of sex acts decreased as gestation increased (Figure 3, Pearson’s correlation coefficient  = −0.322, p = 0.001).

**Table 2:** Sexual behavior by study group.

		Early Pregnancyn (%)	Late Pregnancyn (%)	Postpartumn (%)	Non-Pregnancyn (%)	P value*
**Sex acts in last week**	**0**	7 (14%)	15 (30%)	46 (92%)	11 (22%)	<0.001
	**1**	9 (18%)	15 (30%)	4 (8%)	6 (12%)	
	**2–3**	25 (50%)	13 (26%)	0 (0%)	20 (40%)	
	**>3**	9 (18%)	7 (14%)	0 (0%)	13 (26%)	
**Sex acts in last month**	**0**	2 (4%)	4 (8%)	31 (62%)	2 (4%)	<0.001
	**1–4**	10 (20%)	23 (46%)	17 (34%)	14 (28%)	
	**>4**	38 (76%)	23 (46%)	2 (4%)	34 (68%)	
**Last sex act in days** ∫	**1–7**	44 (92%)	35 (74%)	4 (8%)	38 (79%)	<0.001
	**8–30**	4 (8%)	8 (17%)	20 (40%)	8 (17%)	
	**>30**	0 (0%)	4 (9%)	26 (52%)	2 (4%)	

* Fisher’s exact.

∫ Columns that do not add up to the total are due to missing values.

The difference in sexual activity between groups was corroborated with information from individual participants about changes in their own lives comparing pregnant/postpartum versus non-pregnant times. Most women (62%, 123/199) reported decreased sex acts during pregnant or postpartum periods, as compared to non-pregnant periods. When participants were asked to quantify this change, non-pregnant weekly sex acts exceeded pregnant weekly sex acts by an average of 1.55 acts (p<0.001).

### Risky Behavior

We used several measures of risky behavior, including condom use, STI prevalence, other sex partners, and spouse’s activity, such as nights spent away from home and alcohol use. Most women (68.5%, 137/200) reported never using condoms, although 60% (38/63) of those who use condoms reported more use during non-pregnant times compared to pregnant or postpartum times. The use of condoms during the last 5 sex acts differed across groups with substantially less use in late pregnancy and postpartum (Table 3, p = 0.024).

**Table 3:** Risky behavior by study group.

		Early Pregnancyn (%)	Late Pregnancyn (%)	Postpartumn (%)	Non-Pregnancyn (%)	P value*
**Condom use during**	**0**	40 (80%)	43 (86%)	46 (92%)	36 (72%)	0.024
**last 5 sex acts**	**1–4**	9 (18%)	4 (8%)	1 (2%)	11 (22%)	
	**5**	1 (2%)	3 (6%)	3 (6%)	3 (6%)	
**STI in last 3 months**	**Yes**	3 (6%)	2 (4%)	1 (2%)	0 (0%)	0.519
	**No**	47 (94%)	48 (96%)	49 (98%)	50 (100%)	
**Husband with STI**	**Yes**	1 (2%)	3 (6%)	1 (2%)	3 (6%)	0.600
**in last 3 months**	**No**	45 (90%)	39 (78%)	43 (86%)	47 (94%)	
	**Don’t know**	4 (8%)	8 (16%)	6 (12%)	0 (0%)	
**Times husband drank**	**0**	39 (78%)	39 (78%)	40 (80%)	34 (68%)	0.517
**alcohol in last month**	**1–4**	7 (14%)	4 (8%)	6 (12%)	11 (22%)	
	**>4**	4 (8%)	7 (14%)	4 (8%)	5 (10%)	
**Nights husband came home**	**0**	26 (52%)	32 (64%)	26 (52%)	30 (60%)	0.352
**late in last month**	**1–4**	23 (46%)	15 (30%)	18 (36%)	16 (32%)	
	**>4**	1 (2%)	3 (6%)	6 (12%)	4 (8%)	
**Nights husband went out**	**0**	36 (72%)	36 (72%)	41 (82%)	41 (82%)	0.503
**in last month**	**1–4**	8 (16%)	10 (20%)	8 (16%)	6 (12%)	
	**>4**	6 (12%)	4 (8%)	1 (2%)	3 (6%)	
**Nights husband spent away**	**0**	39 (78%)	37 (74%)	40 (80%)	43 (86%)	0.743
**from home in last month**	**1–4**	5 (10%)	8 (16%)	6 (12%)	5 (10%)	
	**>4**	6 (12%)	5 (10%)	4 (8%)	2 (4%)	

* Fisher’s exact.

The reported STI prevalence over the last 3 months among women and their spouses was similar at 3% (6/200) and 4% (8/182), respectively. Neither STI prevalence in men nor in women was associated with pregnancy status (Table 3). None of the women reported having any sex partners other than their spouse.

Spouse’s risky behavior over the prior month, as measured by the wife’s report, did not differ by pregnancy status for any measure (Table 3). Nights spent away from home, nights out, late return to home, and alcohol use were similar across groups. Of note, 69% (138/200) of women reported that their spouse never drinks alcohol.

When asked about change in the spouse’s risky behavior over time, less than 20% (ranging from 11.5 to 18% depending on the activity) of women noticed any change. Most women that reported change stated that the frequency of nights spent away from home, nights out, and late return to home *increased* (53% vs. 56.5% vs. 61%, respectively) during pregnant and/or postpartum times as compared to non-pregnancy. The change in alcohol use was the opposite with 57% (16/28) of women reporting *less* spousal use during pregnancy/postpartum as compared to non-pregnant times. Although there was no significant difference between the study groups, most individual women who reported change in their spouse’s behavior noticed more risky behavior during pregnancy or postpartum.

### Beliefs about Abstinence

During February 2010, 20 women from the Bwaila clinics completed in-depth interviews. 10 women were enrolled from the antenatal clinic, 3 from the postnatal clinic, and 7 from the family planning clinic. The women had similar demographics to those who completed the quantitative questionnaire (Table 1).

The most consistent belief among the participants was that sex should cease between the 6^th^ and 8^th^ month of pregnancy and not resume again until 6 months postpartum. Beliefs are passed down orally from female elders to new mothers although the reasons for abstaining are often not known or well understood. Women expressed several fears associated with abstaining, including the possibility of spousal infidelity and HIV infection. A 24 year old pregnant woman said:

“People say when one is pregnant sex should stop on the 7^th^ month, but I think that…is not good because it’s like you are forcing the husband to go outside and seek other sexual partners, and at the end you find that one of you catches a disease [HIV].”

Other women were worried about consequences if they did not follow the tradition of abstinence. A 24 year old pregnant woman with two children said:

“For me after delivery, 6 months has to go before I have sex, I totally refuse and I don’t care how my husband thinks or says because I feel my life is precious and if I do something stupid, it is me who is going to be hurt.”

The women talked about the conflict that exists between the tradition of abstinence and their fears of infidelity and HIV. It seems that some beliefs are changing in response to the HIV epidemic as voiced by this woman:

“What I got from my counselors is that in the past people used to say that when you are 6 months pregnant, you must stop having sex with your husband…but now with the HIV pandemic, they recommend that you continue doing it but not so frequent because a man cannot wait for you from 6 months until you give birth to make love to you.”

Several women implied that their generation requires new rules for behavior because men lack self control during times when abstinence is traditionally expected. If infidelity occurs, some women recognized the possibility of their spouse acquiring HIV. One woman spoke about these concerns:

“Stopping sex when one is pregnant is not good because…men and women are different, men can’t stay for so long without sex so if you don’t sleep with them they will be forced to go seek other sexual partners…men of this generation cannot wait until 6 months [after the baby is born].”

While there is some recognition that behavior change is needed, tradition continues to have a profound influence as highlighted by the women who continue to follow cultural beliefs about abstinence despite the possibility of HIV.

## Discussion

HIV incidence is high among pregnant women in Lilongwe at 4 cases per 100 person years. Although sexual activity decreases as gestation advances, a subgroup of women remain sexually active at moderately high levels. Most women report no sexual activity in the postpartum period. The apparent discrepancy between high HIV incidence and fewer sex acts during pregnancy might be explained by behavioral risk, biological risk, or both. Participants reported low rates of condom use overall, and these rates declined further during late pregnancy and postpartum. Other measures of risky behavior, including the behavior of their spouses, did not differ between pregnant, non-pregnant, and postpartum women. However, qualitatively, women expressed fear of increased spousal infidelity during pregnancy.

The incidence of HIV during pregnancy in our study is consistent with other reports from the region. The incidence rate is substantially higher than the 1.4 cases/100 person years reported in a similar, non-pregnant population in Lilongwe [13] and is less than the rate of 7.9 cases/100 person years found in a pregnant population in 1998 [14] (adult HIV prevalence in Malawi has decreased since 1998 from 13.8% to 11.0%) [15]. Our incidence also parallels high incidence during pregnancy found in nearby countries including South Africa (10.7 cases/100 person-years) [5], Zimbabwe (4.8 cases/100 person-years) [1], and Uganda (2.3 cases/100 person years) [2]. Notably, some studies have reported that pregnancy does not increase HIV incidence. Pregnancy was not associated with increased HIV risk in a nested study in Zimbabwe, Zambia, and South Africa [16] and HIV incidence among pregnant women in a study from Uganda and Zimbabwe was not increased [17].

Our HIV incidence rate was calculated by matching HIV results and demographic variables between antenatal and labor ward visits so as to ensure we were comparing the same woman in both settings. However, inconsistent data entry in written registries, such as the spelling of names and the recording of ages, was a challenge. Therefore, all potential seroconversion events may not have been identified and some women may have been mistakenly matched through this process. The matching process serves as an important limitation of our estimate of HIV incidence during pregnancy. Such limitations are not uncommon when working in a healthcare system with limited resources where data is recorded in written registries rather than electronic records.

Sexual behavior change during pregnancy is particularly important in understanding high HIV incidence. In West Africa, female sexual activity decreases during pregnancy and postpartum and men find other sex partners during those periods [6], [8], [9]. Less is known about sexual behavior during pregnancy in Southern Africa. In Zimbabwe, risk of HIV seroconversion in pregnant women increases four fold in those who know their partner has other partners [18] and married women who abstain from sex after delivery are 3.2 times more likely to contract HIV than those who do not abstain [19]. We need to understand the nuances of sexual behavior because it is likely that some married women who say they are “abstaining” continue to have infrequent sex, which explains how they are able to acquire HIV.

In our study, sexual activity significantly declined during late pregnancy and postpartum. While the decline in sexual activity was evident in the overall population, a subgroup of women clearly remain sexually active, even late into pregnancy. Although the frequency of sexual activity is reduced, these women remain at risk for HIV acquisition, particularly since condom use is low during late pregnancy. Qualitatively, women reported that sexual activity should cease between the 6^th^ and 8^th^ month of pregnancy and not resume until 6 months postpartum. This corroborates with a recent study about postpartum abstinence in Malawi reporting that the median time to resumption of sexual activity after childbirth was between 7 and 9 months [20]. In our study, reports of spouse’s risky behavior did not differ between groups. However, most individual women who noticed change over time stated that risky behavior increased during pregnancy or postpartum. In the in-depth interviews, women expressed fears about spousal infidelity during pregnancy, raising the possibility that male infidelity may exist even though our quantitative measures did not detect an increase in risky behavior. In future studies, it will be important to more directly assess male sexual behavior during pregnancy.

Whether pregnant women’s susceptibility is biological, behavioral, or both, they are being exposed to HIV in a way that is increasing their acquisition despite decreased sexual activity. Female risky behavior reportedly does not increase during pregnancy [2], [5]. In this study, pregnant women denied having any sex partners other than their spouse, did not have an increase in STI prevalence, and engaged in less sexual activity as gestation increased. If pregnant women are not engaging in risky behavior, then HIV transmission is likely resulting from increased biological susceptibility (with transmission between a discordant couple) and/or from an acutely infected male spouse with high viral load [21].

The extent to which male behavior change, female biological susceptibility, and decreased condom use impact HIV acquisition during pregnancy are unclear and need to be further studied. CCR5 expression increases as gestation advances, which may contribute to pregnant women’s risk of acquiring HIV during vaginal intercourse [22]. In Uganda, Gray, et al. calculated an increase in HIV acquisition per coital act in pregnant women compared to non-pregnant/non-lactating women without any accompanying change in risky behavior, which suggests a biological susceptibility during pregnancy [2]. The decrease in condom use during pregnancy and postpartum, described in this and prior studies [2], [5], [17], may also contribute to incident cases during pregnancy.

The cross-sectional, mixed methods portion of the study allowed us to compare current sexual behavior between groups of women, to inquire about change over time in individual women, and to learn about the cultural context of behavior change. This design allowed us to corroborate trends in our data. For example, we confirmed that sexual behavior decreases during late pregnancy and postpartum by comparing activity between groups of women as well as calculating change over time in individual study participants. One limitation of our study population was that our non-pregnant cohort was a unique group of women who were seeking contraception to prevent unintended pregnancy at the family planning clinic. Therefore, they may engage in different sexual behaviors than the non-pregnant population as a whole.

Our study found that pregnancy inspires sexual behavior change among women in Lilongwe, oral traditions strongly influence behavior change, and those changes lead to fear of spousal infidelity in some women. Certainly, male partners are affected by the changes in their pregnant wives’ sexual behavior and it is imperative to understand how male behavior changes so efforts can be better targeted to reduce HIV incidence during pregnancy. Although generalizing our findings to other populations must be done cautiously, our data highlights the importance of understanding the cultural context of sexual behavior.

PMTCT programs exist throughout sub-Saharan Africa with the goal of identifying pregnant women infected with HIV and preventing new infections in order to eliminate transmission to infants. Our findings highlight the importance of repeat HIV testing in PMTCT programs in order to detect seroconverters and prevent transmission to the infant during pregnancy or postpartum [23], [24]. Children of pregnant or lactating mothers with incident HIV infection have 2.3 times higher risk of HIV infection [25]. To prevent this transmission, we need to further study behavior change, cultural beliefs about sex, and biological susceptibility during pregnancy. Ultimately, the goal is to develop public health interventions for antenatal and postnatal populations so that we can prevent HIV acquisition and improve healthcare for both mothers and children. As a start, pregnant women should be informed about their increased risk for HIV during pregnancy and the importance of using condoms throughout pregnancy and the postpartum period. Relaying those messages, while we continue to investigate why pregnant women are at greater risk for acquiring HIV, could help to prevent many new cases of HIV.
